# Physical Activity and Health-Related Quality of Life Among Community Dwelling Elderly

**DOI:** 10.14740/jocmr2307w

**Published:** 2015-09-25

**Authors:** Hadeel Halaweh, Carin Willen, Anna Grimby-Ekman, Ulla Svantesson

**Affiliations:** a Department of Physiotherapy, Institute of Neuroscience and Physiology at Sahlgrenska Academy, University of Gothenburg, Sweden; b Health Metrics at Sahlgrenska Academy, University of Gothenburg, Sweden

**Keywords:** Healthy aging, Physical activity, Habitual walking, Comorbidity

## Abstract

**Background:**

Physical activity (PA) and health-related quality of life (HRQoL) are important factors for optimal health in the elderly. Studying the association between PA and HRQoL is becoming more essential as the number of elderly people increases worldwide. This study assesses the association between PA and HRQoL among community dwelling elderly above 60 years old.

**Methods:**

The study included 115 women and 61 men (mean age: 68.15 ± 6.74 years) recruited from the community and from public centers for the elderly. Data were collected using a background characteristics questionnaire (BCQ), a physical activity socio-cultural adapted questionnaire (PA-SCAQ), and the EuroQuol-5Dimensions-5Levels (EQ-5D-5L) questionnaire. Between groups, comparisons were based on the PA-SCAQ by dividing the participants into three PA groups: low (n = 74), moderate (n = 85), and high (n = 17). Kruskal-Wallis tests were performed on the ordinal variables of the three PA groups to determine differences between the groups according to categorical variables such as gender, body mass index (BMI), and the prevalence of comorbid conditions. Mann-Whitney U tests were performed on the ordinal variables of the EuroQuol-5Dimensions (EQ-5D), and the independent sample *t*-test was performed on the EQ visual analogue scale (EQ-VAS). Spearman’s rank correlation coefficient was used to examine the correlation between the EQ-5D and level of PA.

**Results:**

Values in all dimensions of HRQoL were significantly higher (P < 0.05) in the moderate and high PA groups compared with the low PA group. Significant correlations were recorded between the five dimensions of HRQoL and the level of PA (P < 0.001). The low PA group showed higher prevalence of hypertension (64%, P < 0.001) and diabetes (50%, P < 0.001).

**Conclusion:**

There were strong associations between higher levels of PA and all dimensions of HRQoL. Therefore, adopting a PA lifestyle may contribute to better HRQoL among community dwelling elderly above 60 years old.

## Introduction

Participation in physical activity (PA) promotes healthy aging and plays an important role in improving quality of life (QoL) among elderly [1-3]. Evidence suggests that participating in regular moderate intensity PA (e.g., walking, cycling, or light sports) has significant benefits for health, including improved treatment of many diseases [4-6]. The evidence of health benefits of PA is stronger for adults 65 years old and older than for any other age group, since the consequences related to inactivity are more severe for this age group. Compared to less active elderly, active elderly have lower rates of all-cause mortality and a higher level of muscular fitness [1, 7]. Therefore, increasing PA among elderly persons has become an international priority [1]. This priority coincides with the reality that worldwide the number of elderly is increasing and the proportion of people aged over 60 years is growing faster than any other age group. This means society will need to reconsider how to care for elderly in order to maximize the health and functional capacity of elderly persons.

Aging, health status, PA, and disability all affect independence and QoL in elderly persons [6, 8, 9]. QoL is related to an individual’s perception of the position in life in the context of culture and value systems and is influenced in a complex way by the person’s physical health, psychological state, level of independence, and social relationships [10]. Health-related quality of life (HRQoL) is part of a multidimensional approach that considers physical, mental, and social aspects [2]. Assessing HRQoL is an essential component of healthcare evaluation [11]. Several instruments have been developed to assess HRQoL in different populations. Factors related to demographic and clinical characteristics including age, health status, culture, and language of the studied population are important determinants for using a relevant valid and reliable HRQoL measure [11-13].

Higher levels of PA appear to be associated with better functioning and better HRQoL [2, 3, 8, 9, 14]. PA can help older adults reclaim or maintain a healthy aging process [9]. Adopting a physically active lifestyle, including increasing leisure time PA, may result in a better long term HRQoL among elderly persons [15, 16]. Relatively regular moderate PA can help elderly prevent a decline in HRQoL and even improve their enjoyment of life [17]. Since these are important factors that promote optimal health in elderly, studying the association between PA and HRQoL is becoming more essential, especially as the number of elderly is increasing. This study assesses the association between PA and HRQoL in community dwelling elderly (≥ 60 years old).

## Methods

### Participants

A volunteer sample of 176 community dwelling elderly Palestinians from the West Bank was recruited (115 women and 61 men; mean age: 68.15 ± 6.74). Inclusion criteria consisted of women and men aged ≥ 60 years and independence in ambulation with or without walking aids (independence in ambulation was determined based on the participant’s walking capacity, if they were able to walk for at least 6 m independently). Exclusion criteria for the elderly included diagnosis of a severe disease that would make investigations impossible and communication deficits such as could not answer questions about age, children, and current location, time, season, and year.

In this cross-sectional study, all participants were informed about the aim of the study and signed an informed consent. The study received ethical approval from the research ethics committee of Al-Quds University, Palestine (Ref No. 1/REC/13), and the study complies with the Declaration of Helsinki.

### Measurements

#### Background characteristics questionnaire (BCQ)

Demographic clinical descriptive data on age, gender, educational level, job status, smoking habits, medication, diagnosed disease (cardiovascular, musculoskeletal, hypertension, and others), and sensory functions (visual, hearing and speech) were registered. Anthropometric measurements (weight and height) were also recorded.

#### Measure of activities of daily living (ADL)

The Katz index was used to assess personal ADL [18]. The index has been described as a valid and reliable measure to determine independency level in performing ADL [19]. The assessment is based on the ability to perform an activity without assistance from another person. The Katz index of ADL includes six basic activities of daily living (BADL): bathing, dressing, toileting, transferring, continence, and feeding. The ability in performing each activity was assessed using a two-point categorical scale: 1 = independence and 0 = dependence. The total score ranged from 0 (low function, dependent) to 6 (high function, independent).

#### Measure of PA

A physical activity socio-cultural adapted questionnaire (PA-SCAC) was used. The PA-SCAQ includes the following questions: How often do you take outdoor walks? How long are your walks? What household activities and other activities (e.g., yard work gardening) do you do? The structure of the questionnaire concerning PA domains, duration, and frequency was built on the WHO global recommendations [1] as well as by considering some culturally applicable items of the valid PA measures for elderly persons [20, 21]. Accordingly, PA variables were categorized into walking, household activities, and outdoor activities. To compute the accumulated frequency and duration of all the activities, the minutes spent per week on each of these activities were summed. The participants were categorized into three groups based on their moderate-intensity aerobic PA (MIA-PA) (walking) throughout the week: low PA (less than 150 min/week), moderate PA (between 150 and 300 min/week), and high PA (more than 300 min/week).

To assess whether the questionnaire’s questions and the response categories were understandable by and appropriate for the participants, the PA-SCAQ was pilot tested with 10 community dwelling elderly Palestinians with different levels of education (six women and four men between 62 and 83 years old). All questions and response categories were considered comprehensible, so the piloted version was not subjected to any additional modifications and was used in this study as PA-SCAQ.

#### Measure of QoL: the Arabic version of EuroQuol-5Dimensions-5Levels (EQ-5D-5L)

The EQ-5D-5L is a standardized, non-disease specific instrument developed for describing and valuing HRQoL. The instrument has been translated into most major languages including Arabic [12, 22]. The EuroQuol-5Dimensions (EQ-5D) has been described as a valid and reliable instrument to assess HRQoL in different populations [23-25].

The Arabic (Jordan) EQ-5D version was used to measure QoL among the elderly in this study [12]. In March 2013, permission to use the validated Arabic version was obtained from the EuroQoL group. The EQ-5D-5L consists of a descriptive system and the EQ visual analogue scale (EQ-VAS). The descriptive system includes five dimensions (mobility, self-care, usual activities, pain/discomfort, and anxiety/depression) and each dimension includes five levels of coding: 1 = no problems; 2 = slight problems; 3 = moderate problems; 4 = severe problems; and 5 = extreme problems. In addition, respondents reported self-rated health on an EQ-VAS with endpoints labeled 100 (the best health you can imagine) and 0 (the worst health you can imagine).

### Statistical analysis

Data were analyzed using the Statistical Package for the Social Sciences (SPSS) package, version 20 (SPSS Inc., Chicago, IL). Descriptive statistics were used to characterize the sample according to PA and HRQoL variables. Kruskal-Wallis tests were performed on the ordinal variables of the PA groups’ three levels to determine differences between the groups according to categorical variables such as gender, body mass index (BMI), and the prevalence of comorbid conditions. Mann-Whitney U tests were performed on the ordinal variables of the EQ-5D. The independent sample *t*-test was performed on the EQ-VAS to determine differences between women and men and between the three physical activity groups (low, moderate, and high). Spearman’s rank correlation coefficient was used to examine the correlation between EQ-5D and level of PA. Statistical significance was set at P < 0.05.

## Results

The mean age of the participants was 68.15 ± 6.74 years (range: 60 - 91 years), and 76.6% of the participants lived with their families and 23.4% of the participants lived alone. About 42% of the participants had less than 6 years of education. The majority of the participants (92%) were fully independent in the BADL, and 8% were partially independent according to the Katz index. The majority of the participants (86.4%) recorded a diagnosed disease such as hypertension (48.9%), musculoskeletal (54.0%), and cardiovascular disease (22.2%). Clinical characteristics of the participants in relation to comorbidity are illustrated in Table 1 and Table 2.

**Table 1:** Clinical Characteristics of the Participants

Clinical characteristics	Women (n = 115)	Men (n = 61)
	Mean (SD)	Mean (SD)
Weight (kg)	78 (14.7)	82 (11.27)
Height (cm)	156 (0.06)	170 (0.06)
Body mass index	32 (5.09)	28 (3.84)
Diagnosed disease	n (%)	n (%)
Yes	101 (88)	51 (84)
No	14 (12)	10 (16)
Cardiovascular	22 (19)	17 (28)
Diabetes	36 (31)	18 (30)
Hypertension	57 (50)	29 (48)
Hyperlipidemia	25 (22)	5 (8)
Musculoskeletal	70 (61)	25 (41)
Osteoporosis	28 (24)	2 (3)
Sensory function		
Visual problems	83 (72)	44 (72)
Hearing problems	23 (20)	7 (12)
Taking medications	95 (83)	50 (82)
Been hospitalized in the last year	28 (24)	11 (18)
Regular medical checkup	63 (55)	34 (56)
Using assistive devices		
Glasses	66 (57)	43 (71)
Hearing aids	3 (3)	1 (2)
Cane	12 (10)	12 (20)
Walks duration (min/week)		
< 150	57 (50)	17 (28)
150 - 300	52 (45)	33 (54)
> 300	6 (5)	11 (18)

SD: standard deviation; n: number of cases.

**Table 2:** Clinical Characteristics Among Different Physical Activity Groups

Variables	Low PA group (n = 74)	Moderate PA group (n = 85)	High PA group (n = 17)	P
Gender (female/male (n))	57/17	52/33	6/11	0.001
BMI (kg/m^2^) (mean (SD))	32 (5.1)	30 (4.9)	29 (5.0)	0.029
Diagnosed disease (%)	97	81	65	< 0.001
Cardiovascular (%)	31	17	12	0.01
Diabetes (%)	50	22	6	< 0.001
Hypertension (%)	64	44	12	< 0.001
Osteoporosis (%)	31	7	6	< 0.001
Hyperlipidemia (%)	24	13	6	0.072

Values are presented as percentages, or mean (SD). SD: standard deviation; BMI: body mass index; n: number of cases.

Descriptive values of PA variables showed that 50% of the women and 28% of the men walked less than 150 min/week, which is also the definition of the low PA group in the present study (Table 1). In addition, 17% of the women and 79% of the men spent less than 150 min/week doing household activities, 69% of the women and 6% of the men spent more than 300 min/week doing household activities, and 70% of the women and 46% of the men spent less than 150 min/week participating in outdoor activities.

Results of HRQoL measures showed that 64% of the participants recorded problems walking, ranging from slight problems to moderate and severe problems. In addition, 68.7% of the participants indicated that they had problems doing their usual activities. The majority of the participants (about 80.1%) recorded having pain or discomfort ranging from slight to extreme pain. No significant differences were recorded according to gender on all HRQoL dimensions except on the mobility dimension. Men recorded a mobility median score of 1 (I have no problem in walking about) and women recorded mobility median score of 2 (I have slight problems in walking about) (Table 3).

**Table 3:** Health-Related Quality of Life (HRQoL) According to Gender

HRQoL variables	Women (n = 115)	Men (n = 61)	P
Mobility, n (%)			
I have no problems in walking about	34 (30)	31 (51)	
I have slight problems in walking about	59 (51)	19 (31)	
I have moderate problems in walking about	15 (13)	9 (15)	0.030
I have severe problems in walking about	7 (6)	2 (3)	
I am unable to walk about	0 (0)	0 (0)	
Self-care, n (%)			
I have no problems washing or dressing myself	65 (57)	37 (61)	
I have slight problems washing or dressing myself	27 (23)	13 (21)	
I have moderate problems washing or dressing myself	19 (16)	10 (16)	0.583
I have severe problems washing or dressing myself	3 (3)	1 (2)	
I am unable to wash or dress myself	1 (1)	0 (0)	
Usual activities (e.g. work, study, housework, family or leisure activities), n (%)			
I have no problems doing my usual activities	37 (32)	18 (29)	
I have slight problems doing my usual activities	32 (28)	20 (33)	0.850
I have moderate problems doing my usual activities	36 (31)	15 (25)	
I have severe problems doing my usual activities	7 (6)	8 (13)	
I am unable to do my usual activities	3 (3)	0 (0)	
Pain/discomfort, n (%)			
I have no pain or discomfort	18 (15)	17 (28)	
I have slight pain or discomfort	55 (48)	28 (46)	0.057
I have moderate pain or discomfort	30 (26)	11 (18)	
I have severe pain or discomfort	10 (9)	5 (8)	
I have extreme pain or discomfort	2 (2)	0 (0)	
Anxiety/depression, n (%)			
I am not anxious or depressed	30 (26)	20 (33)	
I am slightly anxious or depressed	52 (45)	29 (48)	0.108
I am moderately anxious or depressed	14 (12)	10 (16)	
I am severely anxious or depressed	18 (16)	2 (3)	
I am extremely anxious or depressed	1 (1)	0 (0.0)	
EQ-VAS (mean (SD))	70.4 (13.40)	75.8 (14.38)	0.543

SD: standard deviation; HRQoL: health-related quality of life; n: number of cases.

Significant differences between the three PA groups were recorded on the five dimensions of EU-QoL and EQ-VAS, where the higher PA groups recorded higher scores in all EU-QoL dimensions (Table 4). Significant correlations were also recorded between the five dimensions of (EQ-5D-5L) and level of PA in terms of walking minutes (P < 0.001) (Table 5).

**Table 4:** Health-Related Quality of Life (HRQoL) Measures Among Physical Activity (PA) Groups

HRQoL variables	Low PA group (n = 74)	Moderate PA group (n = 85)	High PA group (n = 17)	P_1_	P_2_
Mobility, n (%)					
I have no problems in walking about	11 (15)	41 (48)	13 (76)		
I have slight problems in walking about	33 (45)	42 (49)	3 (18)		
I have moderate problems in walking about	21 (28)	2 (3)	1 (6)	< 0.001	< 0.001
I have severe problems in walking about	9 (12)	0 (0)	0 (0)		
I am unable to walk about	0 (0)	0 (0)	0 (0)		
Self-care, n (%)					
I have no problems washing or dressing myself	25 (34)	64 (75)	13 (77)		
I have slight problems washing or dressing myself	16 (22)	20 (24)	4 (23)		
I have moderate problems washing or dressing myself	28 (38)	1 (1)	0 (0)		
I have severe problems washing or dressing myself	4 (5)	0 (0)	0 (0)	< 0.001	< 0.001
I am unable to wash or dress myself	1 (1)	0 (0)	0 (0)		
Usual activities (e.g. work, study, housework, family or leisure activities), n (%)					
I have no problems doing my usual activities	13 (18)	31 (36)	11 (65)		
I have slight problems doing my usual activities	14 (19)	33 (39)	5 (29)		
I have moderate problems doing my usual activities	33 (44)	17 (20)	1 (6)		
I have severe problems doing my usual activities	11 (15)	4 (5)	0 (0)		
I am unable to do my usual activities	3 (4)	0 (0)	0 (0)	< 0.001	< 0.001
Pain/discomfort, n (%)					
I have no pain or discomfort	10 (13)	20 (23)	5 (29)		
I have slight pain or discomfort	27 (36)	49 (58)	7 (42)		
I have moderate pain or discomfort	22 (30)	14 (16)	5 (29)	< 0.001	0.034
I have severe pain or discomfort	13 (18)	2 (2)	0 (0)		
I have extreme pain or discomfort	2 (3)	0 (0)	0 (0)		
Anxiety/depression, n (%)					
I am not anxious or depressed	15 (20)	27 (32)	8 (47)		
I am slightly anxious or depressed	27 (37)	46 (54)	8 (47)		
I am moderately anxious or depressed	17 (23)	6 (7)	1 (6)	0.001	0.002
I am severely anxious or depressed	14 (19)	6 (7)	0 (0)		
I am extremely anxious or depressed	1 (1)	0 (0)	0 (0)		
EQ-VAS (mean (SD))	63 (13.51)	78 (9.74)	85 (9.43)	< 0.001	< 0.001

P_1_: comparison between low and moderate PA groups; P_2_: comparison between low and high PA groups.

**Table 5:** Correlation Between HRQoL Variables and PA Level

Variable	r_s_	95% confidence interval	
		Lower	Upper
HRQoL variables			
Mobility	-0.630	-0.669	-0.418
Self-care	-0.526	-0.614	-0.352
Usual activities	-0.536	-0.617	-0.356
Pain/discomfort	-0.353	-0.458	-0.174
Anxiety/depression	-0.381	-0.479	0.197
EQ visual analogue scale	0.657	0.463	0.706

r_s_: Spearman’s correlation coefficient; HRQoL: health-related quality of life; EQ: Euro Quality.

## Discussion

### Methodological consideration

Determining a precise PA measurement to be used for elderly persons is very difficult due to physiological and cognitive changes that occur with aging [26]. This challenge can be more intense in a circumstance where the available valid measures for assessing PA in elderly [20, 21] cannot be entirely applied according to different domains of PA within diverse cultural contexts [1]. Therefore, this study uses an adapted questionnaire that considers social and cultural issues. The PA-SCAQ was constructed based on the WHO recommendations on PA, but it also considers some culturally applicable items of the valid PA measures for elderly persons [20, 21]. The prevalent domain of PA among the elderly Palestinians is more revolved around activities such as walking, gardening, and household chores [1]. Findings of the PA-SCAQ pilot testing corresponded with these more common activities. Thus we believe the PA-SCAQ had good face validity and enabled us to obtain the required descriptive statistics about PA domains among the elderly.

We used the data obtained from the PA-SCAQ to determine PA levels. The WHO [1] recommends that elderly persons should do at least 150 min of MIA-PA throughout the week. However, for additional health benefits, increasing MIA-PA to 300 min per week is recommended. The MIA-PA is defined as “activity in which the body’s large muscles move in a rhythmic manner for a sustained period of time. Examples include walking, running, swimming, and bicycling” [1]. In our study, MIA-PA was typically obtained through walking, so levels of PA were determined using walking duration. Walking is one of the most common PAs among elderly persons and can easily be adapted into daily life [27]. Daily walks for at least 30 min have shown to be positively related to leg muscle strength and self-rated physical fitness among elderly [28].

The two major domains of interest for studying HRQoL in this study were functioning and subjective well-being [29]. To evaluate functioning, we used the EQ-5D-5L to determine mobility, self-care, and usual activities. To evaluate subjective well-being, we used the EQ-5D-5L to determine pain/discomfort, anxiety/depression, and VAS. Furthermore, the EQ-5D-5L is a valid measure for Arabic speaking elderly [12]. EQ-5D-5L was found to be a practical and simple tool for elderly populations [23, 24]. Because about 42% of the participants in this study had less than 6 years of education, using EQ-5D-5L helped achieve a good response rate.

A possible limitation in this study was that the PA-SCAQ determines an overall description of PA level based on the walking minutes without quantifying various types of PA, an issue that was challenging to address within the scope of the present study.

### Results’ discussion

In this study, women constituted 77.0% of the low PA group and the majority of the participants (86.4%) reported having at least one diagnosed disease. The low PA group recorded higher prevalence of chronic diseases such as cardiovascular, hypertension, and diabetes. Moderate intensity activities such as walking, gardening, or light sports appear to have beneficial effects and lead to a 30-50% reduction in cardiovascular disease in women [4]. Several studies have shown that regular moderate intensity of PA such as walking has significant benefits for health and for the treatment of a number of diseases [3, 5, 6]. Accordingly, increasing PA among elderly persons has become an international priority [1], so the elderly have been encouraged to regularly participate in moderate intensity activities [6, 9], culturally and age appropriate PA interventions for elderly are necessary to be established [1].

Higher PA groups recorded better values in all EQ-5D-5L dimensions. In the functioning domain, compared with the low PA group, the moderate and the high PA group recorded better scores on mobility (P < 0.001). Women recorded lower scores on the mobility dimension of the HRQoL, and they constituted the majority of the low PA active group, findings that support the association between level of PA and HRQoL. This result might be attributed to the fact that more elderly women than elderly men are homebound. Because women are more likely to spend time performing household activities, their mobility level in relation to walking outdoors may be affected. Frandin et al found [28] that walkers had a more positive attitude towards PA as well as a higher estimation of their own physical fitness than non-walkers.

The high PA group recorded higher values for self-care and the usual activities represented in the EQ-5D. Hence, self-care and usual activities affect independence and a higher PA level contributes to reduced risk of disability in elderly [30]. Therefore, this study evaluated the association between PA levels and these components of the functioning domain of EQ-5D.

We addressed the subjective well-being domain of HRQoL using data about pain/discomfort and anxiety/depression as well as reported self-rated health on the EQ-VAS. The state of well-being refers to an individual’s feelings at the time they are expressed [31]. Almost half of the participants indicated having slight pain or discomfort and were slightly anxious or depressed. Significant differences were recorded on the variables of the subjective well-being domain with a significantly higher VAS score compared to the lower PA group. These findings are consistent with similar studies that have shown the protective effect of PA on depression and bodily pain among elderly [3, 32].

### Conclusion

There were strong associations between higher levels of PA and all dimensions of HRQoL. The high PA group showed better values in all dimensions of HRQoL. In addition, the prevalence of comorbid conditions was higher in the low PA group. Therefore, adopting a physically active lifestyle may contribute to better health and HRQoL among the elderly.
